# Occurrence of urinary schistosomiasis and associated bacteria in parts of Ondo State, Nigeria

**DOI:** 10.1371/journal.pgph.0001119

**Published:** 2022-10-12

**Authors:** Kikelomo J. Kone, Anthony K. Onifade, Ebenezer O. Dada

**Affiliations:** 1 Department of Biological Sciences, University of Medical Sciences, Ondo, Ondo State, Nigeria; 2 Department of Microbiology, Federal University of Technology, Akure, Ondo State, Nigeria; COMSATS University Islamabad, PAKISTAN

## Abstract

Schistosomiasis is a parasitic disease caused by blood flukes (trematode worms) of the genus *Schistosoma* and is common among the rural community dwellers that have occupation or recreation activities that link them with infected water bodies. The disease wreak a lot of havoc in the victims which range from anaemia, increase risk of liver fibrosis and bladder cancer, enlarged liver, difficult and painful urination, infertility etc. Nigeria has been reported to be the most endemic country in the world for schistosomiasis with about 29 million infected cases. However, people with urinary schistosomiasis are vulnerable to secondary infections caused by bacteria as a result of the break down in the mucosa barrier occasioned by the wear and tear of the spiny eggs of the schistosomes. Meanwhile, the control measures instituted by various agencies against schistosomiasis paid little attention to its co-infection with bacteria. This study was designed to evaluate the occurrence of urinary schistosomiasis and co-infection with bacteria in parts of Ondo State, Nigeria. Ethical approval was obtained from the Ethical Committee of the Ondo State Ministry of Health, Akure. Macroscopic and microscopic examinations, and microbiological analysis of the urine samples collected were performed using standard techniques. Of the five hundred and nine (509) urine collected, ova of *Schistosoma haematobium* were detected in one hundred and twenty one 121 (23.77%), significant bacteriuria was detected in 104 (20.43%) and co-infection was detected in thirty six 36 (29.75%) of schistosomiasis cases. There was a positive association between urinary schistosomiasis and bacteriuria (X^2^(1) = 8.481, *p* = 0.004). This study revealed a high occurrence of urinary schistosomiasis and significant bacteriuria in the study areas which suggests that bacterial presence may be a potent complication in the management of urinary schistosomiasis.

## Introduction

Schistosomiasis is the second most common socio-economically devastating parasitic disease after malaria [1], affecting about 240 million residents of developing countries. Schistosomiasis is an acute and chronic disease caused by blood flukes (trematode worms) of the genus *Schistosoma*. [2] reported that estimates showed that at least 220.8 million people required preventive treatment in 2017, while [3] reported that 90% of the over 200million infected people live in sub-Saharan Africa.

Nigeria has been reported to be the most endemic country in the world for schistosomiasis [4] and *S*. *haematobium* is widely spread mainly in riverine areas and communities around impoundment of river (dam) [5]. Schistosomiasis is common among poor communities that are without clean potable water and proper sanitation. Schistosomiasis is caused by different species of *Schistosoma* which can be intestinal and urinary depending on the species that is responsible. *S*. *mansoni*, *S*. *japonicum*, *S*. *menkongi* and *S*. *intercalatum* are all responsible for intestinal schistosomiasis while *S*. *haematobium* is responsible for urinary schistosomiasis. Water snails of *Bulinus* species are the intermediate host for *S*. *haematobium* and *S*. *intercalatum* while *Biompheleria* species are the intermediate hosts for *S*. mansoni, *Oncomelania is for S*. *japonicum and for S*. *mekongi* is *Neotricula aperta* [6].

Report by [7] posited it that about 29 million infected cases of schistosomiasis are in Nigeria with 101 million people at risk of infection [8] as there is unabated increase of infection in all the geographical zones of the country. Meanwhile, the urinary tract is the second commonest site after respiratory tract for bacterial infection [9] with over 150 million cases per year [10]. Urinary tract infection (UTI) has been pointed to be the second most frequent infection in long-term care facilities and the most common cause of hospitalization for bacterial infection, [11]. In addition, [12] reported that UTIs are most common outpatient infections, with a lifetime incidence of 50-60% in adult women. Urinary tract infection is as a result of presence of significant bacteria in urine and significant bacteriuria is defined as a urine sample containing more than 10^5^ colonies/ml of urine in pure culture [13].

Bacterial infections in most cases complicate the course of patients with urinary schistosomiasis because the otherwise so-called normal flora of the urinary tract have a way of entering and invading the underlying internal tissues due to the regular wear and tear of the epithelium by the spiny schistosoma eggs [14]. Therefore, associated bacterial infections have been suggested to be the major pathogenetic elements in schistosomiasis, rather than parasitic effects [15].

Co-infections of urinary schistosomiasis and urinary tract infections (UTIs) caused by *Schistosoma haematobium* and bacteria respectively are very common among children in the tropics [16] described UTI as a pandemic disease that its incident is greatly influenced by age and sex and by factors that impair the defense mechanism that maintain the sterility of the normal urinary tract.

Many studies have implicated bacteriuria co-infection with urinary schistosomiasis in the aetiology of bladder cancer and other complications [9,15]. UTI is responsible for more illnesses and significantly contribute to the cost of providing health globally, leading to a number of deaths either from acute infection or from chronic renal failure. More so, bacterial infections normally take place, when the mucosal barrier is broken down which makes the urinary tract an easy target for invading bacteria [17].

Despite the evidence of possible association of bacteruria with urinary schistosomiasis, the control measures instituted by various agencies against schistosomiasis pay little attention to the complexity of schistosomiasis morbidity in the presence of UTI. This however, could be due in part to the dearth of information on the association between these parasitic and bacterial urinary infections.

This study set out to evaluate the prevalence of schistosomiasis in parts of Ondo State, Nigeria and investigate the prevalence of co-infection of schistosomiasis and UTI.

## Materials and methods

### Description of study area

The study was conducted among the dwellers of the selected communities viz: Ayede Ogbese in Akure North Local Government Area, Ita-Oniyan and Aponmu in Akure South Local Government Area and Ipogun in Ifedore Local Government Area of Ondo State Nigeria. The selection of these communities was based on information from the Ondo State Primary Health Care Development Agency that urinary schistosomiasis were common in the areas. They are rural communities that their major occupation is farming but Ogbese is more civilized among the four communities being a community that is densely populated by people from different parts of the country and engage in trading in addition to farming activities. The trading activities attract people from various cities and different states of Nigeria. Apart from farming, their women are also involved in local production of palm oil which makes them to have regular contact with the *Schistosoma* infested rivers; this is also common to the women in the other three communities. The economic activities of the people in these communities includes fishing, palm-oil production and production of food and tree crops like cocoa, oil-palm, cashew, rubber, teak, gmelina and different species of indigenous trees. All the four communities under study have rivers and streams that pass through them. River Ogbese is found in Ogbese, River Awo in Ita-Oniyan links up with Aponmu community and River Aponmu is in Ipogun links up with Aponmu community. The community dwellers use the rivers and streams as their sources of water for domestic and recreational purposes.

### Ethics statement

Ethical approval was obtained from the Ethical Committee of the Ondo State Ministry of Health, Akure. Formal consent was obtained in both written and verbal forms. Informed consent forms were administered to the people and the literate ones among them filled and signed the forms while verbal consent of the illiterate ones were obtained for them to participate after the content of the form was explained to them. Only those that agreed to participate by filling and signing the forms and those that consented verbally were given sterile universal bottles for urine collection.

### Collection and analysis of urine samples

Convenience sampling method was employed for this study and the sample collection was carried out in between September 2018 and October 2019 and the collection cut across both rainy and dry seasons. Participants were educated on collection of clean catch urine with emphasis on the last drop after which sterile universal bottles were given to them. Females were asked to clean their genitalia before passing the urine into the sterile bottles. Urine samples were collected between 10.00 a.m. and 2.00 p.m. and transported to the laboratory in ice pack within 1hr of collection. A total of five hundred and nine (509) urine samples were collected from the communities, three hundred and seven (307) and two hundred and two (202) in the rainy (between May and October) and dry (between November and April) seasons respectively. All the samples were examined macroscopically by checking the appearance and 10 mls of each of the urine was introduced into clean centrifuge tube for spinning. The urine samples were centrifuged at 2500 revolution per minute (rpm) for 10 mins after which the sediments were placed on grease free glass slides, covered with cover slips and examined for ova of *Schistosoma haematobium* under a microscope with X10 to check for presence of any ova and later X40 objective lenses for higher magnification and clarity of the ova that were seen.

### Microbiological analysis of the urine samples

Urine samples were cultured using filter paper dip strips as described by [18]. Blood agar and Cystein Lactose Electrolyte Deficient (CLED) media were used for the primary isolation of bacteria present in the urine samples. All the plates were incubated at 37°C for 18-24 hr after which the plates were examined for bacterial growth. Samples with bacterial growth of ≥ 10^5^ colony forming unit (CFU) per ml of urine were noted and considered positive. All the bacteria isolates were sub-cultured on nutrient agar to get pure isolates. The bacteria isolates were identified using morphological characteristics by checking for colour or pigment production, shape, elevation, surface, consistency, size and edges of the colonies. Gram’s staining procedures, reactions on special media and series of biochemical tests with un-inoculated tubes as control [19] were equally performed.

### Data analysis

Data generated was entered into Microsoft excel spreadsheet of version 2010 and later exported into the Statistical Package for Social Sciences (SPSS) version 23 for analysis. Results were presented in form of tables and charts. Bivariate Pearson Chi-square was used to determine the association between schistosomiasis and each of gender, season and location. Also, association between UTI and each of gender, season and location was examined using bivariate Pearson Chi-square and statistical significance was established at 0.05 level of significance. Association between urinary schistosomiasis and UTI was determined using correlation analysis.

## Results

**Fig 1:**
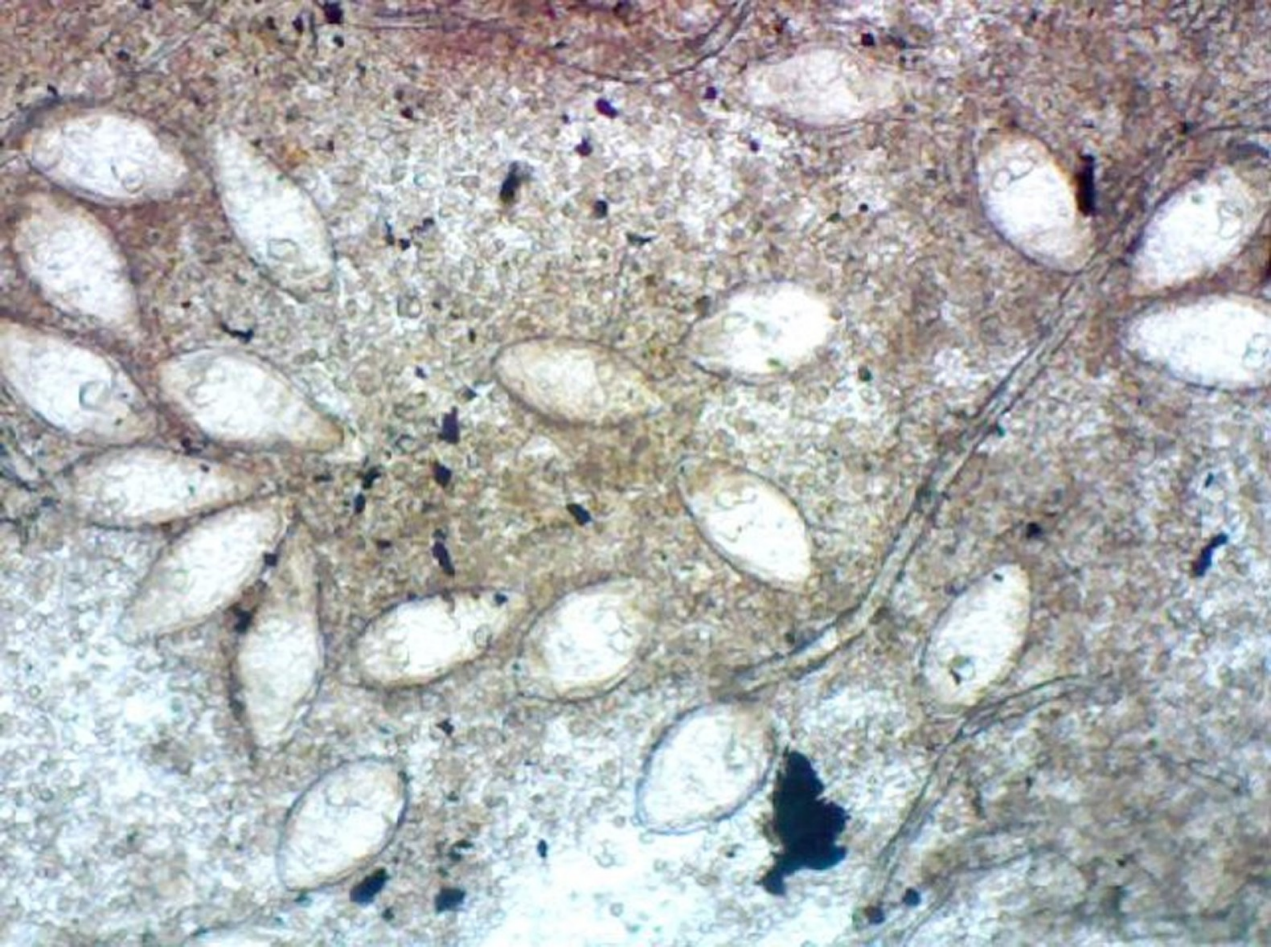
Ova of *S*. *heamatobium* x400.

Of the five hundred and nine (509) participants, two hundred and forty-six (246) representing 48.33% were females. Three hundred and thirty three (333) of the urine samples appeared to be clear; one hundred and forty two (142) were cloudy, while thirty four (34) appeared bloody. One hundred and twenty one (121) samples contained ova of *Schistosoma haematobium* representing 23.77% of the entire samples examined (Fig 1) and (Table 1).

**Table 1:** Occurrence of infections in relation to communities and seasons.

		LOCATION					SEASONS		
VARIABLES	Sig. Diff	AP	IP	IT	OG	Sig. diff.	D	R	TOTAL
Schistosomiasis		20	30	42	29		73	48	121
%		23.81	28.04	29.37	16.57		60.33	39.67	23.77
	P value	0.036				P value	0.000		
Bacteriuria		31	23	30	20		68	36	104
%		36.90	21.50	20.98	11.43		65.38	34.62	20.43
	P value	0.000				P value	0.000		
Co-infection		8	12	9	7		25	11	36
%		9.52	11.21	6.29	4.00		69.44	30.56	7.07
	P value	0.102				P value	0.000		
Total		84	107	143	175		202	307	509

P < 0.05.

Key: IP - Ipogun, OG - Ogbese, IT - Ita-oniyan, AP - Aponmu, D - dry season, R - rainy season.

Furthermore, one hundred and four (104) representing 20.43% of the total samples showed significant bacteriuria comprising (20) Ogbese, (23) Ipogun, (31) Aponmu and (30) Ita-Oniyan respectively (Table 1). Aponmu community had the highest UTI occurrence of 36.9% while Ogbese had the lowest (11.43%). The overall co-infection was 7.07% with Ipogun having the highest percentage of co-infection (schistosomiasis and bacteriuria) at 11.21% (Table 1). Both schistosomiasis and UTI together with their co-infection were season-dependent as they were all significant at *p* = 0.000 which was less than the *p*-value of 0.05. All the infections were more prevalent in dry season as compared to rainy season (Table 1).

*Enterobacter aerogenes*, *Enterococcus faecalis*, *Escherichia coli*, *Klebsiella pneumoniae*, *Proteus vulgaris*, *Pseudomonas aeruginosa*, *Serratia marcescens*, *Staphylococcus aureus* and *S*. *saprophyticus* were presumptively the isolated bacteria. *S*. *aureus* had the highest percentage occurrence and the most implicated cause of co-infection with schistosomiasis while *K*. *pneumoniae* and *E*. *faecalis* were the least cause of co-infection with schistosomiasis (Table 2).

**Table 2:** Bacteria isolates, frequency and co-infection.

Bacteria isolates		Frequency	Percent	Co-infection (%)
	*E*. *coli*	19	3.7	5 (13.89)
	*E*. *faecalis*	1	.2	1 (2.78)
	*E*. *aerogenes*	2	.4	0 (0.00)
	*K*. *pnuemoniae*	7	1.4	1 (2.78)
	*P*. *aeruginosa*	11	2.2	5 (13.89)
	*P*. *vulgaris*	2	.4	0 (0.00)
	*S*. *aureus*	36	7.1	13 (36.11)
	*S*. *saprophyticus*	25	4.9	11 (30.56)
	*Serratia marcescens*	1	.2	0 (0.00)

The Occurrence of schistosomiasis was higher in males 86/121 than in females at 35/121, while that of bacteriuria was higher in females 55/104 than in males at 49/104 (Table 3). However, sex-specific occurrence of schistosomasis was significant (X^2^(1) = 23.934, *p* = 0.000), bacteriuria was not significant (X^2^(1) = 1.086, *p* = 0.297) while the total occurrence of the co-infection was significant (X^2^(1) = 4.901, *p* = 0.027).

**Table 3:** Occurrence of the individual infections and co-infection in relation to gender.

		Sig. diff	UTI (%)		Sig. diff	Schistosomiasis (%)		Sig. diff	Co-infection (%)	
SEX			Yes	No		Yes	No		Yes	No
	Female		55 (22.4)	191 (77.6)		35 (28.9)	211 (54.4)		11 (30.6)	235 (49.7)
	Male		49 (18.6)	214 (81.4)		86 (71.1)	177 (45.6)		25 (69.4)	238 (50.3)
		P. value	0.297		P. value	0.000		P. value	0.027	
Total			104 (204)	405 (96)		121 (23.7)	388 (76.23)		36 (7.07)	473 (92.93)

P < 0.05.

Similarly, sex-specific occurrence rate of bacteriuria was more apparent in females at 52.88% than in males at 47.12%. Total occurrence of the co-infection was higher in males than females at 70.27% and 29.73% respectively. Ipogun had the highest percentage of co-infection (schistosomiasis and bacteriuria) at 12.15% while Ogbese had the least at 4.46%. Aponmu and Ita-Oniyan had 9.52% and 6.29% respectively.

Occurrence rate of schistosomiasis was more during the dry season at 46.43% compared to 20.51% occurrence during the rainy season in Ipogun. Similarly, higher occurrence rate was observed during the dry season at 41.67% compared to 7.09% occurrence during the rainy season in Ogbese. Likewise, higher schistomiasis occurrence was observed in Aponmu and Ita-Oniyan communities at 27.27% and 33.8% during dry season compared to concurrent 17.24% and 23.61% prevalence during the rainy season respectively.

Occurrence rate of bacteriuria was more during the dry season at 39.29% compared to 15.38% occurrence during the rainy season in Ipogun. Similarly, higher occurrence was observed during the dry season at 49.09% compared to 13.79% occurrence rate during the rainy season in Aponmu. Likewise, higher bacteriuria occurrence was observed in Ita-Oniyan and Ogbese communities at 33.8% and 18.75% through the dry season compared to concurrent lower 12.5% and 8.66% rate of occurrence during the rainy season respectively.

The highest tendency for co-infection during the rainy season was observed in Ipogun at 6 (7.6) while the least was observed at Aponmu and Ita-Oniyan respectively at 1 (3.4) and 1 (1.4). The highest tendency for co-infection of schistosomiasis and bacteriuria during the dry season was observed in Ita-Oniyan at 8 (11.3) while the least was observed in Ogbese at 4 (8.3).

Generally, season specific occurrences of schistosomiasis, bacteriuria and the co-infection were significant (X^2^(1) =28.265), *p* = 0.000), (X^2^(1) = 36.064, *p* = 0.000) and (X^2^(1) = 14.333, *p* = 0.000) respectively.

## Discussion

The occurrence of urinary schistosomiasis and associated bacteria in urine subjects in parts of Ondo State, South-West, Nigeria was ascertained in this study. Few studies in respect of urinary schistosomiasis have considered all ages and limited studies have compared co-infection of schistosomiasis and bacteriuria. This was attributed to the belief that schistosomiasis was common in certain age groups which informed the treatment decision recommended for school age children. The bloody and cloudy appearance of many of the urine called for attention. This was in agreement with the report of [20], that cloudiness in freshly voided urine is clinically significant. The abnormal appearance was an indication that people that voided the urine samples could have diseased conditions. The occurrence rate of urinary schistosomiasis in the study area (23.77%) was high compared to 8.3% reported by [21] in the study conducted in the northern part of the country. This could be attributed to lack of mass chemotherapy of praziquantel, the drug of choice for schistosomiasis prior to the period of this study. The occurrence rate was low compared to 44.8% and 71.5% in Osun and Ogun States, respectively as reported by [22]. This could be attributed to attitidunal change of the people due to the health education by the Ministry of Health officers known to implement the annual mass praziquantel administration.

The higher occurrence of schistosomiasis at Ita-Oniyan (29.37%), Ipogun (28.04%) and Aponmu (23.81%) might be as a result of linkage of the communities by the rivers (Aponmu and Awo). The three communities were interconnected with the rivers as compared to Ogbese (16.57%) that was far from them. Majority of the residents of these communities relied on the schistosomes-infested rivers for their household chores as they were short of other sources of clean water like borehole, well, tap water etc. High rate of occurrence of schistosomiasis in males was in conformity with some existing reports [1,9,23,24]. This might be due to anthropogenic activities like swimming, fishing, sporting, washing and bathing in the water. There was a relationship between the occupation and gender of the participants in Ogbese community as all the positive cases for schistosomiasis were males. The majority of the females that were traders and female farmers among them did not have frequent contact with the river.

Recreational activities in river favoured the proliferation of schistosomiasis and, by extension, the co-infection. From this research, occurrence of schistosomiasis was dependent on season with high rate of occurrence in dry season compared to rainy season. This might be due to more frequent visits to the rivers in dry season than rainy season. Water scarcity and the need to cool off due to high heat generated during the day could increase the people’s frequent visits to the water bodies. More so, Ondo State is known for high temperature in the dry season due to high effect of the sun and little would one wonder why the State is termed “Sunshine State”. Abundance of *Bulinus globosus*, the snail host of *S*. *haematobium* depends on velocity of the water because if the velocity is high, water sweeps the snails away and in dry season, the velocity of water is low which might be responsible for the increase in the snail population and the occurrence of urinary schistosomiasis in the areas [25]. This fact was corroborated by Partin [26] that reported that *Bulinus* snails prefer slow-flowing fresh-water habitats and are able to withstand low oxygen conditions. In addition, the flooded river bank might discourage regular visits to the streams in rainy season. The people might also have other sources of water during rainy season that prevented them from visiting the rivers during the season. Socio-cultural beliefs of the people could be another reason for the low occurrence of schistosomiasis in rainy season [27].

High occurrence rate of UTI among the participants was in consonance with the results of [9] and [28] which could still be linked to the infected water and abuse of antibiotics as the people paid regular visit to patent medicine stores instead of the basic health centers in the communities.

Though the number of female participants with UTI was more than that of male but the difference was not significant. The wider surface area of female genitalia and the shortness of the urethra of the female urinary tract could predispose them to UTIs than men [10], and this should have made the female participants to be more prone to UTI rather than males. High occurrence of *S*. *aureus* and *S*. *saprophyticus* in the urinary tract was in agreement with the report of [29,30]. This could be due to their association with skin and affinity for wet area with high level of salt. However, the higher occurrence of *S*. *aureus* bacteriuria should be of a great concern as reported by [31] that it could be a sign of a serious infection, like *S*. *aureus* bacteremia or an occult abscess.

This report did not conform to the reports from [32] that posited *E*. *coli* as the predominant bacteria encountered in their study. For *P*. *aeruginosa* and *Serratia marcescens* opportunistic pathogens to be found in urinary tract might be due to the debilitating conditions of the people in these communities. The occurrence rate of co-infection (7.07%) recorded in this study was low compared to 53.7% reported by [9]. This could be as a result of the people’s patronage to local chemists using antibiotics to ‘treat’ schistosomiasis. Co-infection of schistosomiasis and UTI been higher in males could be due to their immune-compromised state.

## Conclusion

Findings from this study revealed a high rate of occurrence of urinary schistosomiasis and UTI in the study areas. In addition, *S*. *aureus* was implicated as the most frequently encountered bacteria in urine from the study. Also, it was discovered that occurrence rate of schistosomiasis was more in dry season than rainy season. This study clearly suggests that UTI is a potent complication in the management of urinary schistosomiasis. Hence the complimentary incorporation of antibacterial regimen in schistosomiasis control is essential.

## Supporting information

S1 Data Occupation represents the job description of the participants and it ranges from farming to artisan like automobile mechanic, tailoring/fashion designing, welding, hairdressing, bricklaying, palm oil production, studentship, civil/public service. **The applicants** represent the job seekers, business depicts those that were into buying and selling while the apprentice were those into acquisition of one skill or another among the participants. **Educational background** represents the highest level of formal education attained by the participants which ranged from kindergarten (pre-nursery) to nursery, primary, grade II teacher certificate, modern school (no longer inexistence), secondary education. **The higher and national diplomas, first and second university degrees** represent tertiary education. **None** depicts those without any formal education at all. **Knowledge of UTI and schistosomiasis** highlights the participants’ pre-knowledge about urinary tract infection (UTI) and schistosomiasis respectively. **Bloody urine** depicts if the participants have passed bloody urine in one time or another as it is one of the pointer for urinary schistosomiasis. **Contact with water** explains if the participants indulged in any activity that linked them with the infected water. **Waist pain** depicts the participants experienced waist pain/lower abdominal pain as it is one of the symptoms of UTI. The expected response was **“Yes” or “No”**. **Painful urine** has to do with whether the participants were experiencing pain while **passing urine** as this is one of the symptoms of UTI. **Passage through water** explains whether or not the participants do pass/walk through the infected water while going to their farmlands, schools, place of work or from one community to another. **The duration**, ranging from minutes to hours, depicts the length of body contact with the infected water. Presence or absence of ova of ***S*. *haematobium*** was depicted by “Yes” or “No” respectively. The column for **bacteria** signifies whether or not significant bacteria cells were discovered in the urine samples after culturing them. **Type of bacteria** explains the identity of the bacteria based on the results generated from biochemical tests. **Appearance** depicts the macroscopic appearance of the urine as viewed with the naked eyes, while **location** describes the communities where the samples were collected. **IP**- represents Ipogun in Ifedore Local Government area, **OG**- Ogbese in Akure North Local Government **IT**- Ita-Oniyan and **AP**- Aponmu, both in Akure South Local Government area of Ondo State. **The Season** represents the period of the year when the urine samples were collected. **D**- stands for dry season which is normally experienced between November and April, while **R**- stands for rainy season and this usually occur between May and October of every year.
(XLSX)
